# Subjektive Belastung der Eltern durch die Beschulung ihrer Kinder zu Hause zu Zeiten des Corona-bedingten Lockdowns im Frühjahr 2020

**DOI:** 10.1007/s11618-021-01012-9

**Published:** 2021-04-01

**Authors:** Sabine Zinn, Michael Bayer

**Affiliations:** 1 SOEP am DIW, Berlin, Mohrenstraße 58, 10117 Berlin, Deutschland; 2 Humboldt-Universität zu Berlin, Berlin, Deutschland; 3 Leibniz-Institut für Bildungsverläufe e. V. (LIfBi), Bamberg, Wilhelmsplatz 3, 96047 Bamberg, Deutschland; 4 Evangelische Hochschule Nürnberg, Nürnberg, Deutschland

**Keywords:** Subjektive Belastung, Beschulung zu Hause, Corona-bedingter Lockdown, Alleinerziehende, Niedriger Bildungsabschluss, Corona-related lockdown, Home schooling, Subjective stress, Low educational attainment, Single parents

## Abstract

Die Corona-bedingten Schulschließungen sowie die Schließung von Kinderbetreuungseinrichtungen im April und Mai 2020 haben viele Eltern vor eine immense Herausforderung gestellt. Plötzlich mussten Kinder ganztags Zuhause betreut und beschult werden. In diesem Beitrag beschäftigen wir uns mit der Frage nach der subjektiven Belastung, der sich Eltern durch die Beschulung ihrer Kinder Zuhause ausgesetzt sahen. Hierbei legen wir ein besonderes Augenmerk auf die individuelle Ressourcenausstattung der Eltern sowie auf ihre familiäre Situation und ihr Erwerbsleben. Insbesondere untersuchen wir das subjektive Belastungsempfinden alleinerziehender Eltern. Für unsere Analysen nutzen wir die Daten der SOEP-CoV Studie, einer Sonderbefragung an Panelteilnehmern des Sozio-Oekonomischen Panels (SOEP) zum Thema Corona. Insgesamt konnten wir bei allen befragten Eltern (*N* = 1508, davon *N* = 243 alleinerziehend) eine mäßige Belastung durch die Anforderungen der Beschulung ihrer Kinder Zuhause ausmachen. Besonders belastet fühlten sich jedoch Eltern mit einem niedrigen Bildungsabschluss und alleinerziehende Eltern, insbesondere wenn sie zur Zeit der Schulschließungen erwerbstätig waren. Unsere Analysen legen nahe, dass gerade diese Elterngruppen Probleme hatten, den Anforderungen, die eine Beschulung Zuhause mit sich bringt, unter den gegebenen Umständen umfassend nachzukommen.

## Abstract (en)

The Corona-related school closures and the closure of childcare facilities in April and May 2020 meant an immense challenge for many parents. Suddenly, children had to be looked after and educated at home all day. In this paper, we address the question of the subjective burden that parents faced when schooling their children at home. In doing so, we pay special attention to the individual resources of the parents as well as to their family situation and their working life. In particular, we examine the subjective perception of stress of single parents. For our analyses we use data from the SOEP-CoV study, a special survey of the Socio-Economic Panel (SOEP) on the topic of Corona. Overall, we found moderate levels of stress from the demands of schooling their children at home among all parents surveyed (*N* = 1508, of which *N* = 243 were single parents). However, parents with a low level of education and single parents felt particularly burdened, especially if they were employed at the time of the school closures. Our analyses suggest that precisely these groups of parents had problems comprehensively meeting the demands of home schooling under the given circumstances.

## Einleitung und theoretische Herleitung

### Kollektive Erfahrung der vollständigen Schulschließung

Dieser Beitrag beschäftigt sich empirisch mit der Frage nach dem subjektiven Belastungsempfinden, dem sich Eltern durch die Corona-bedingten Schulschließungen und den damit verbundenen Beschulungsaufgaben ausgesetzt sahen. Hierfür werden die Daten einer für die Eltern von Schulkindern repräsentativen Sonderstudie des Sozio-Oekonomischen Panels (SOEP) genutzt.

Die Corona-Pandemie hat im März 2020 zur vollständigen Schließung aller Schulen in Deutschland geführt. Dieses Ereignis kann als kollektive Erfahrung oder ein kollektives kritisches Lebensereignis (zum Konzept kritischer Lebensereignisse vgl. Filipp und Ammans 2018) angesehen werden. Eine derartige breite Betroffenheit durch ein singuläres Ereignis findet sich ansonsten nur in Kriegs- bzw. Terrorsituationen (vgl. Collins 2004), bei globalen Umweltkatastrophen (vgl. hierzu und zu der damit verknüpften Diskussion über die Logik der Risikoverteilung Beck 1986), beim Zusammenbruch ganzer Gesellschaften oder in Situationen umfassender wirtschaftlicher Einbrüche bzw. wirtschaftlicher Depression (vgl. Elder 1999). Entsprechend selten ergibt sich die Gelegenheit Menschen bereits während solcher Ereignisse zu befragen und damit deren Wahrnehmungen und Empfindungen ohne den Filter einer retrospektiven Einschätzung zu erheben.

Solche gesellschaftsweiten Ereignisse manifestieren sich jedoch letzten Endes auf der Ebene der Individuen und müssen dort individuell bzw. partnerschaftlich oder auch in Gruppen verarbeitet werden. Die damit verbundenen konkreten Reaktions- und Verarbeitungsnotwendigkeiten sind in erheblichem Maße von den Ressourcen und Verarbeitungsstrategien der verschiedenen Menschen oder Gruppen abhängig. Auch wenn derartige Ereignisse einen gewissen singulären Charakter besitzen, lassen sich hieraus durchaus Einsichten gewinnen, die es ermöglichen die Nutzbarkeit von theoretischen Modellen zu prüfen, die für weniger disruptive Situationen und Prozesse entwickelt wurden. Darüber hinaus verfolgt die nachstehende Analyse vor allem aber auch das Ziel, die empirischen Befunde im Hinblick auf die vorhandenen gesellschaftspolitischen Implikationen zu diskutieren. Dies ist auch insofern relevant, als dass bezüglich nachgelagerter Lockdown-Ereignisse, wie z. B. den Corona-bedingten Lockdown zum Jahresende 2020 und am Jahresbeginn 2021, Lehren hinsichtlich institutioneller Unterstützungsbedarfe und langfristiger Folgen z. B. mit Blick auf eine potenzielle Verstärkung von Bildungsungleichheiten gezogen werden können.

Das COVID-19 Snapshot Monitoring (COSMO)^1^ sammelt bereits seit Anfang März 2020 wöchentlich Informationen zu Wahrnehmungen und Einschätzungen der Bevölkerung im Hinblick auf die Bedrohung durch das Virus. Außerdem trägt COSMO Informationen zu den Maßnahmen zusammen, welche durch die Regierungen und Behörden beschlossen und umgesetzt wurden und werden. Mit den Daten dieses querschnittlich angelegten Monitorings lässt sich zeigen, dass die Befürwortung von *Schulschließungen* zwischen dem 10. und 17. März sprunghaft zugenommen hat, über ca. 2–3 Wochen auf hohem Niveau verblieb und bis in die zweite Junihälfte nach und nach wieder auf das Niveau Anfang März zurückging (https://projekte.uni-erfurt.de/cosmo2020/web/). Bezieht man in diese Ergebnisse die Befunde des Nationalen Bildungsberichts ein, dass 68 % aller Schulen im Sekundarbereich im Schuljahr 2018/19 ganztägig organisiert waren und rund die Hälfte aller Grundschulkinder ein Angebot von entweder schulischer Ganztagsbetreuung oder Betreuungsangeboten durch Kindertageseinrichtungen in Anspruch nahm (Autorengruppe Bildungsberichterstattung 2020, S. 119 ff.), dann wird deutlich, dass mit den Schulschließungen im März 2020 gleichzeitig erhebliche Veränderungen im Bereich der außer- und innerhäuslichen Betreuung einhergingen, die jedoch – zumindest in den ersten Wochen der Schulschließungen – auf Basis einer breiten öffentlichen Zustimmung stattgefunden haben. Wie genau jedoch die davon betroffenen Eltern diese allgemein befürworteten Schulschließungen tatsächlich verarbeiten, ist nicht zuletzt von der familialen Situation und den dort vorhandenen Ressourcen und Strategien abhängig.

Im Zuge der vollständigen Schließung aller Schulen in Deutschland sind es insbesondere Fragen zu den damit einhergehenden Auswirkungen auf die Bildungschancen, die im Zentrum bildungsbezogener Diskussionen stehen (Anger und Plünnecke 2020). Da die Phase der Schulschließungen für die Bildungsentwicklung der Schülerinnen und Schüler nicht ungenutzt bleiben sollte, wurden die Schulen durch die Kultusministerien der Länder angehalten, wenn möglich digitale Überbrückungsangebote zu entwickeln, bei denen sie in unterschiedlicher Weise durch die Ministerien unterstützt wurden (vgl. hierzu https://www.kmk.org/themen/bildung-in-der-digitalen-welt/lernen-von-zu-hause-digitale-lernangebote.html). Gleichzeitig wurde Ende März 2020 in Abstimmung zwischen Bund und Ländern beschlossen, Mittel aus dem „DigitalPakt Schule“ kurzfristig für die Krisenbewältigung einzusetzen und somit die digitalen Möglichkeiten der Schulen zu erweitern. Sowohl die Schulschließungen selbst wie auch diese Ad-hoc-Digitalisierung der Schulen inklusive damit verbundener Möglichkeiten und Risiken stand von Anfang an im Fokus der Ungleichheitsforschung (vgl. etwa van Ackern et al. 2020). Auch die dritten Ad-Hoc-Stellungnahme der Nationalen Akademie der Wissenschaften (Leopoldina) am 13. April 2020 wies auf mögliche Probleme diesbezüglich hin (https://www.leopoldina.org/publikationen/detailansicht/publication/leopoldina-stellungnahmen-zur-coronavirus-pandemie-2020/).

Welche mittel- und längerfristigen Auswirkungen die durch die Corona-Pandemie hervorgerufenen Schulschließungen auf die Ungleichheitsstruktur des Bildungsbereichs haben wird, lässt sich derzeit noch nicht abschließend beantworten. Dass entsprechende Effekte (vor allem bezüglich der Verstärkung sozialer Ungleichheiten) zu erwarten sind, lässt sich allein durch den Blick auf die diesbezüglich relevanten Studien zum sogenannten Ferien-Effekt plausibilisieren (vgl. für die USA Cooper et al. 1996; für Deutschland Siewert 2013).

### Schulschließung und Beschulung zu Hause als Stressor

Die vollständige Schließung sämtlicher Schulen in Folge der Corona-Pandemie im Frühjahr 2020 stellte eine kollektive Erfahrung für alle Haushalte dar, welche schulpflichtige Kinder haben. Kollektive Erfahrungen besitzen, so wissen wir seit den religionssoziologischen Studien von Emile Durkheim (1994), gepaart mit kollektiven Emotionen, das Potenzial Solidarität zu aktivieren bzw. zu erneuern. Die hohen Zustimmungswerte zu den Schulschließungen zu Beginn der Krise im März 2020 signalisieren, dass in der breiten Öffentlichkeit hinsichtlich der politischen Reaktionen eine ausgeprägte Einigkeit bestand.

Gleichzeitig stellen Schulschließungen generell für alle Betroffenen ein exogenes Ereignis dar, auf welches sie reagieren müssen. Die Schulschließungen im März 2020 bezogen sich letztlich jedoch nur auf den Ort Schule, nicht auf die Beschulung selbst. So ging mit den Schulschließungen eine Umstellung auf kombinierte Formen von Distanzunterricht und elterlicher Unterstützung bzw. elterlicher Kontrolle einher. Unter solchen Bedingungen werden Eltern in einem über das Normale hinausgehenden Ausmaß in die schulbezogenen Aktivitäten ihres Kindes einbezogen. Das subjektive Belastungsempfinden (durch die Schulschließungen) kann hierbei als Maß dafür angesehen werden, ob und inwiefern sich Eltern dieser Herausforderung gewachsen bzw. umgekehrt, in welchem Maße sie sich durch diese Situation überfordert fühlen.

Das von Conger et al. (1990) vorgeschlagene *Family Stress Model *(FSM) beschreibt einen möglichen theoretischen Rahmen für eine derartige Situation. Das FSM beschäftigt sich in seiner ursprünglichen Form insbesondere mit den Auswirkungen von materiellen Deprivationen auf innerfamiliale Prozesse und im Speziellen mit der Herausbildung elterlichen emotionalen Stress, der sich wiederum auf die Zuwendungsbereitschaft aber auch die konkrete Zuwendung zum Kind auswirkt. Die Schließung von Schulen lässt sich im FSM als ein akuter Stressor beschreiben, dem die Ressourcen, die eine Familie aufbringen kann (z. B. das elterliche Bildungskapital oder die Ausstattung der häuslichen Lernumwelt), als ein Indikator für materiellen und – im betrachteten Fall auch immateriellen – Deprivationen gegenüberstehen. Je nach Passungsfähigkeit zu den Herausforderungen können die vorhandenen Ressourcen dann das subjektive Belastungsempfinden durch die Beschulung der Kinder zu Hause entweder steigern oder reduzieren. Der im FSM angelegte psychologische Vermittlungsmechanismus zwischen materieller bzw. immaterieller Situation und kindlicher Entwicklung betont die hohe Relevanz der subjektiven Wahrnehmungen und Verarbeitungen der Lebensbedingungen, in denen sich die Familien befinden und dies auch im Hinblick auf die unterschiedlichen Familienformen (Heintz-Martin und Langmeyer 2020). Somit bietet eine um den Faktor „immaterielle Deprivation“ (wie dem elterlichen Bildungskapital oder der verfügbaren Zeit) erweiterte Version des FSM eine Möglichkeit das subjektive Belastungsempfinden im Lockdown im Frühjahr 2020 zu beschreiben. Die etwa in der Familienform sichtbar werdenden unterschiedlichen Lebensbedingungen lassen sich auch als vorhandene Vulnerabilitäten verstehen, die die Anpassungsfähigkeit an die durch die Schulschließungen ausgelöste Situation in direkter Weise beeinflussen. Dieser Zusammenhang zwischen vorhandenen Vulnerabilitäten, stressauslösenden Ereignissen und daraus resultierenden Anpassungen an die Situation wurde von Karney und Bradbury (1995) als Vulnerabilitäts-Stress-Adaptations-Modell vor allem für die Analyse von daraus resultierenden Beziehungsqualitäten entwickelt, kann jedoch gleichzeitig als eine Erweiterung des FSM vor allem im Hinblick auf nichtmaterielle Vulnerabilitäten verstanden werden. Allerdings ist im vorliegenden Fall der Stressor „Schulschließung“, anders als bei z. B. Zerstörungen durch Naturkatastrophen (siehe hierzu u. a. die Studie von Lowe et al. 2012 zu den Auswirkungen des Hurrikan Katrina unter Verwendung des FSM), durch die grundsätzliche Zustimmung zu der stressauslösenden Maßnahme gefiltert – ein Mechanismus, der so im FSM nicht berücksichtigt ist. Diese Einschränkung muss bei der Anwendung des FSM hinsichtlich der Wirkung familiärer Ressourcen auf das elterlichen Belastungsempfinden beachtet werden.

Subjektives Belastungsempfinden ist jedoch in erheblichem Maße auch von gruppenspezifischen Bezügen abhängig, in welchen sich Eltern befinden bzw. auf die sie in ihren Wahrnehmungen rekurrierten. So führen Tankart und Planck (2016, S. 184) aus: „*Humans are especially motivated to understand and to follow the norms of groups that we belong to and care about, known as reference groups.*“ Ob und inwiefern die Wahrnehmung von und die Einstellung zu Schulschließungen bereits ein gruppenspezifisches Phänomen darstellt und inwieweit also die Zustimmung zu den Schließungen über alle familiären Gruppen streut, lässt sich auf Basis der vorhandenen Daten nicht rekonstruieren.^2^

Auch bedeuten vollständige Schulschließungen die gleichzeitige Betroffenheit unterschiedlicher Lebensbereiche. Entsprechend führen Elcheroth und Drury (2020, S. 2) aus: „*When a population is faced with a major upheaval in their daily lives, the shock may simultaneously affect several, or most, determinants of social behaviour.*“ Zudem sind Haushalte, etwa in Abhängigkeit von der genauen Haushalts- und Beschäftigungsstruktur in unterschiedlicher Weise von derartigen Schulschließungen betroffen.

### Besondere Situation der Alleinerziehendenhaushalte

Insbesondere Alleinerziehendenhaushalte befinden sich u. a. aufgrund des mit dieser Haushaltsstruktur verbundenen höheren Armutsrisikos (vgl. BMAS 2017) auch ohne zusätzliche krisenhafte Einschnitte bereits in einer Situation hoher Belastung (Heintz-Martin und Langmeyer 2020). Insofern liegt die Vermutung nahe, dass gerade diese Haushaltsform in besonderem Maße durch Schulschließungen betroffen ist. Alleinerziehendenhaushalte unterscheiden sich vor allem aber strukturell von Paarhaushalten, wobei zusätzliche Aufgaben, die mit einem erhöhten Betreuungs- und Zuwendungsaufwand für das Kindes einhergehen, in der hier untersuchten Situation auch nicht anderweitig kompensiert werden können, sondern haushaltsintern bewältigt werden müssen. So zeigt sich etwa bei Alleinerziehenden, deren familiärer Status aufgrund einer vorangegangenen Scheidung zustande kommt (nach wie vor die größte Teilgruppe; siehe Peuckert 2019), ein verstärkter Zugriff auf familiäre Ressourcen außerhalb des Haushalts (vgl. etwa Harknett und Knab 2007). Gleichzeitig verfügen Alleinerziehende auch über weniger soziale Kontakte (Cairney et al. 2003). Dies ist nicht nur im Hinblick auf die eingeschränkten Möglichkeiten der Kompensation von Zusatzanforderungen relevant, sondern belegt auch, dass genau diese Gruppe auf die institutionellen (Betreuungs- und Beschulungs‑)Angebote angewiesen ist. U. a. zeigt Keim-Klärner (2020) auf, dass Alleinerziehende hinsichtlich sozialer Kontakte, sozialer Unterstützung wie auch allgemein des vorhandenen sozialen Kapitals im Vergleich zu Ehepaaren deutlich benachteiligt sind.

Neben diesen mit der Haushaltsstruktur zusammenhängenden Unterschieden in den zu vermutenden subjektiven Belastungsempfindungen, die vor allem durch Unterschiede in den sozialen und zeitlichen Ressourcen bedingt werden, die im Sinne des Vulnerabilitäts-Stress-Adaptations-Modells (Karney und Bradbury 1995) die Anpassungsfähigkeit an die neuartige Situation beeinflussen, stellen Schulschließungen auch eine Herausforderung im Hinblick auf inhaltliche Unterstützungsmöglichkeiten dar. Hier ist es dann vor allem der Bildungshintergrund der Eltern, der als einflussreich angenommen werden kann.

### Familiäre Ressourcen

Eltern mit höheren Bildungsabschlüssen stehen im Allgemeinen stärker in Verbindung mit der Schule ihres Kindes. Dadurch sind sie deutlich informierter über die (aktuellen) schulischen Belange ihres Kindes (Lareau 2000). Dies drückt sich auch in einer größeren Beteiligung am schulbezogenen Leben der Kinder aus (vgl. Lareau 2003). Sowohl die größere Nähe zur schulischen Lebenswelt des Kindes wie auch das mit dem eigenen Bildungshintergrund einhergehende bildungsbezogene Wissen sowie das schulische Interesse kann als auswirkungsreich für das subjektive Belastungsempfinden hinsichtlich der mit den Schulschließungen einhergehenden Anforderungssituation angenommen werden. Während die Schulschließungen als solche einen auf alle gleichermaßen wirkenden Belastungsfaktor darstell(t)en, entsteht erst im Zusammenspiel mit individuellen Voraussetzungen bzw. Ressourcen das, was man in stresstheoretischen Modellen als Beanspruchung bezeichnet (vgl. hierzu Böhm-Kasper 2004).

Auch wenn der anhand vorhandener Zertifikate gemessene Bildungshintergrund der Eltern nicht deckungsgleich mit der Informiertheit über die Schule des Kindes bzw. die schulischen Anforderungen wie auch das schulische Interesse der Eltern ist, zeigen Forschungsbefunde jedoch entsprechende Zusammenhänge der Faktoren (vgl. Feldhaus 2015; Hoover-Dempsey et al. 2005). Dementsprechend nutzen wir in unseren Analysen zum Belastungsempfinden der Eltern den (durch Zertifikate gemessene) elterlichen Bildungshintergrund als Proxy sowohl für ihr bildungs- und schulbezogenes Wissen wie auch für ihr schulbezogenes soziales Kapital. Mit dieser Vorgehensweise greifen wir den von Kevin Marjoribanks formulierten Vorschlag auf, der das kulturelle und soziale Kapital als „*educational capital*“ in sein Erklärungsmodell integrierte (vgl. Marjoribanks 2002). In Anknüpfung an die Befundlage zum sogenannten primären Herkunftseffekt (vgl. überblicksartig zu den unterschiedlichen Effekten und Effektkontexten Maaz et al. 2009) ist davon auszugehen, dass das elterliche Bildungsniveau in einer derartigen Krisensituation wie der vorliegenden differentiell kompensierend wirkt – und dies auch und gerade im Hinblick auf die Einschätzung dessen, was die Eltern durch die Schulschließungen zu erwarten hatten. Bezogen auf die vorangegangenen Ausführungen bezüglich des FSM stellt das elterliche Bildungsniveau (als Bildungskapital) somit eine familiäre Ressource dar, die im Hinblick auf Schulschließungen dem akuten Stressor Schulschließung entgegensteht und zu entsprechend differentiellen subjektiven Belastungsempfindungen führt.

Die Erfüllbarkeit bzw. die Wahrnehmung der Erfüllbarkeit von Zusatzaufgaben durch die Schulschließungen, ist (unabhängig vom Bildungsniveau der Eltern) auch von anderen elterlichen Ressourcen abhängig, die sich insbesondere in der Kombinierbarkeit von Erwerbstätigkeit und Bildungs‑/Betreuungsaufgaben zeigt. Dass sich hier auch entlang der Unterscheidung von Paar- und Alleinerziehendenhaushalten deutliche Differenzen, etwa bezogen auf die Möglichkeiten von Home-Office in der Corona-Pandemie zeigen, konnten jüngst Müller u. a. mit Daten des SOEP zeigen (Müller et al. 2020). Blickt man auf die wichtigen Befunde aus Studien zur Nutzung und Wirkung von ganztägiger Beschulung im Hinblick auf die Vereinbarkeit von Erwerbstätigkeit und Familie, so wird deutlich, dass der Wegfall außerhäuslicher Beschulung insbesondere für erwerbstätige Alleinerziehende (die etwa im Bereich der offenen Ganztagsgrundschule überproportional auf ganztägige Angebote zurückgreifen) ein gravierender Einschnitt ist (vgl. Böllert 2020).

### Unterschiedliche kindliche Förderbedarfe und Lernangebote

Nicht außer Acht gelassen werden darf, dass Schulschließungen unter dem Bildungs- und Betreuungsaspekt je nach Alter der Kinder unterschiedlich wirken. Während die Schließung von Grundschulen vor allem im Hinblick auf die Organisation von Betreuung eine Herausforderung für Eltern darstellte, nehmen bildungsbezogene Herausforderungen mit dem Alter der Kinder zu, während zugleich der Betreuungsaspekt in seiner relativen Bedeutung abnimmt.

Auch der Förderaspekt bei der Nutzung ganztägiger Angebote spielt für Eltern aus niedrigeren (Bildungs‑)Schichten neben der Vereinbarkeit von Familie und Beruf eine wichtige Rolle. Böllert (2020) zeigt dies z. B. im Hinblick auf die schichtspezifische Nutzung von Ganztagsangeboten. Insofern ist davon auszugehen, dass die mit zunehmendem Alter der Kinder einhergehende Zunahme an bildungsbezogenen Anforderungen an die Eltern sich in einem bildungsabhängigen Belastungsempfinden ausdrücken, während sich der mit zunehmendem Alter einhergehende geringere Betreuungsaufwand unabhängig von der Bildung der Eltern auf alle gleichermaßen auswirkt.

Mit Blick auf die Möglichkeit zur institutionellen Förderung, die Lernen in der Schule für Kinder mit Migrationsgeschichte oder Migrationshintergrund im Allgemeinen bedeutet, liegt die Vermutung nahe, dass die Eltern solcher Kinder die mit den Schulschließungen einhergehenden Herausforderungen als deutlich belastender empfinden. Allerdings findet sich bei Personen mit Migrationshintergrund die Familienform „Alleinerziehend“ deutlich seltener als bei Personen ohne Migrationshintergrund (vgl. Destatis und WZB 2018, S. 59).

Die mit den Schulschließungen verknüpfte Aufgabenverlagerung in den Bereich der Privathaushalte und insbesondere die Wahrnehmung der damit einhergehenden zusätzlichen Betreuungs- und Kontrollaufgaben, können – über die bereits diskutierten Zusammenhänge hinaus – ebenso als in Abhängigkeit von den durch die Schule initiierten Maßnahmen stehend vermutet werden. Der von den Kultusministerien gewünschte und durch zusätzliche Mittel unterstützte Aufbau von digitalen Distanzunterrichtsstrukturen aber auch die Etablierung von Informationskanälen zwischen Schule bzw. Lehrkräften und Schülerinnen und Schülern können durchaus als ein Faktor verstanden werden, der für Eltern entlastend wirken kann. Der Branchenverband der Deutschen Informations- und Telekommunikationsbranche (Bitkom) zeigt in einer bundesländerspezifischen Zusammenstellung digitaler Lernangebote an deutschen Schulen (https://www.bitkom.org/Themen/Digitale-Unterstuetzung-in-Zeiten-von-Corona/Digitale-Lernangebote-der-Bundeslaender), dass die Schulen in Reaktion auf die Schulschließungen im Zuge der Corona-Pandemie teilweise an bereits vorhandene Lösungen anknüpfen konnten bzw. manche Schulen sich auch in entsprechenden Pilotierungsprojekten befanden, die mit bereits vorhandener digitaler Infrastruktur einhergingen.

### Zentrale Forschungsfragen und Hypothesen

Ausgehend von den skizzierten theoretischen Hintergründen und aufbauend auf vorhandenen Befunden zum Zusammenspiel von Elternhaus, Erwerbstätigkeit und Schule, steht in den folgenden Analysen die Frage im Zentrum: **In welcher Weise beeinflussen vorhandene Ressourcen sowie vorhandene Belastungen das subjektive Belastungsempfinden der durch die Schulschließungen erzeugten zusätzlichen Anforderungen, welche sich vor allem in einer stärkeren Verlagerung von schulbezogenen Aktivitäten in den privaten Bereich zeigen?**

Wir untersuchen diese Frage entlang von Hypothesen, welche sich aus den zuvor diskutierten Überlegungen und Befunden herleiten lassen. Mit den ersten beiden Hypothesen konzentrieren wir uns auf die Gruppe aller Eltern von Schulkindern unabhängig von der jeweiligen Familienform, während die Hypothesen drei und vier sich auf die Gruppe der Alleinerziehenden beziehen.

#### H1:

Wir gehen davon aus, dass Schulschließungen (als akuter Stressor im Sinne des FSM) und damit verbunden der Wegfall einer institutionellen Bildungs- und Betreuungsentlastung insbesondere bei denjenigen Eltern deutliche Auswirkungen auf die Einschätzung der hierdurch ausgelösten Belastungssituation haben, bei denen institutionelle Angebote (sowohl unter einer Betreuungs- wie auch einer Bildungsperspektive) eine hohe Relevanz im Gesamtarrangement darstellen. Dies, so unsere konkretisierte Vermutung, dürfte sich insbesondere im Vergleich zwischen Paarhaushalten und Alleinerziehendenhaushalten dahingehend zeigen, dass Alleinerziehende die Situation im Gruppenvergleich mit Paarhaushalten als deutlich belastender empfinden.

#### H2:

Wir gehen ferner davon aus, dass der Bildungshintergrund der Eltern als eine Form des bildungs- und schulbezogenen Kapitals das subjektive Belastungsempfinden als Reaktion auf die mit den Schulschließungen einhergehenden inhaltlichen Anforderungen als kompensatorische Ressource beeinflusst. Entsprechend vermuten wir, dass unabhängig von der Familienform das Belastungsempfinden durch die Schulschließungen bei einem höheren elterlichen Bildungsniveau geringer ausfällt als bei einem niedrigen elterlichen Bildungsniveau.

#### H3:

In der Gruppe der Alleinerziehenden gehen wir davon aus, dass mit zunehmendem Alter des Kindes ein Entlastungseffekt einhergeht, der unabhängig vom Bildungsniveau des Elternteils das subjektive Entlastungsempfinden reduziert. Diese Hypothese begründet sich in der Annahme, dass für Alleinerziehende die inhaltlichen Herausforderungen durch die Organisation von Schule als Fernunterricht für ältere Kinder relativ geringer sind als die Betreuungsherausforderungen (zeitlich und organisatorisch) für jüngere Kinder.

#### H4:

Wir gehen aber auch davon aus, dass nicht erwerbstätige Alleinerziehende sich, jenseits des vorhandenen bildungsbezogenen Kapitals, in deutlich geringerem Maße durch die Schulschließungen belastet fühlen als diejenigen, welche einer Erwerbstätigkeit nachgehen. Dies, so unsere Vermutung, ist unabhängig davon ob diese Erwerbstätigkeit in Vollzeit oder Teilzeit ausgeübt wird, da die Vereinbarkeitsarrangements von erwerbstätigen Alleinerziehenden nachweislich generell weniger Spielräume beinhalten (z. B. durch ein geringes Maß an Home-Office Möglichkeiten).

#### H5:

Wir vermuten weiter, dass – als stresshemmender Faktor – eine verstärkte Unterstützung durch die Schule durch die Übermittlung von Lernmaterial auf verschiedenen Wegen (z. B. per E‑Mail, Konferenzschaltung, persönlicher Kontakt mit der Lehrkraft etc.) zu einer Verminderung des elterlichen Belastungsempfinden durch die Beschulung ihrer Kinder zu Hause führt, und dies insbesondere bei Alleinerziehenden.

## Daten und Methoden

### Datengrundlage und Stichprobenbeschreibung

Die nachfolgenden Untersuchungen wurden mit Daten der SOEP-CoV Studie durchgeführt, siehe auch http://soep-cov.com. Die SOEP-CoV Studie ist eine telefonische Befragung (CATI) von Haushalten in Deutschland, die auf der Stichprobe des SOEP beruht. Konkret wurden alle Haushalte des SOEP (ohne die Geflüchteten Stichproben und Haushalte, die keine Interviewer gestützte Befragung wünschen^3^ sowie Haushalte ohne valide Telefonnummer) im Zeitraum vom 1. April bis zum 4. Juli 2020 angerufen und jeweils ein erwachsenes Haushaltsmitglied um Angaben zu verschiedenen sozio-ökonomischen Aspekten während der Corona-Zeit gebeten. Insbesondere wurden die Befragungspersonen gebeten über ihre Erfahrungen, die sich durch die politisch induzierten Maßnahmen im Rahmen der Corona-Pandemie in ihrem Alltag ergeben haben, zu berichten.^4^ In diesem Zusammenhang wurden sie nach ihrer Gesundheit und ihrem Gesundheitsverhalten, der Arbeitsmarktsituation und der Erwerbsarbeit sowie nach dem sozialen und dem Familienleben gefragt. Den Befragungspersonen mit Kindern wurden zudem Fragen zur Kinderbetreuung und zur Situation der Beschulung Zuhause gestellt. Insgesamt konnten im Rahmen der SOEP-CoV Studie *N* = 6677 Haushalte befragt werden. Um den zeitlichen Verlauf der Krise und den Verlauf der von der Politik auferlegten Beschränkungen besser abfangen zu können, verlief die Befragung im Rahmen der SOEP-CoV Studie in neun Tranchen, die getrennt in einem Abstand von zwei bis drei Wochen kontaktiert wurden. Das heißt, die Stichprobe ist zufällig auf neun Teilstichproben verteilt worden, die zu unterschiedlichen Zeiten ins Feld gingen. So ist es möglich, die SOEP-CoV Daten dezidiert entsprechend der äußeren Umstände, die gerade zu einem bestimmten Zeitpunkt vorlagen, zu analysieren. In diesem Beitrag interessiert uns im Besonderen die Zeit der bundesweit geltenden totalen Ausgangsbeschränkungen und der Schließung von Schulen und Kinderbetreuungseinrichten. In diese Zeit fallen die Tranchen 1 bis 4 der SOEP CoV Daten. Die zugehörigen Daten wurden im Zeitraum vom 1. April bis zum 30. Mai 2020 erhoben. Insgesamt konnten in dieser Zeit im Rahmen der Studie *N* = 1508 Eltern von Schulkindern befragt werden. Da die Befragungszeit der Interviews beschränkt war, wurden jeweils nur Daten zum jüngsten Schulkind erhoben. Kontextdaten zu weiteren Kindern im Haushalt konnten aus den SOEP Daten 2019 zugespielt werden. Insgesamt wurden *N* = 1244 Eltern mit mindestens einem weiteren Erwachsenem im Haushalt (nachfolgend als Eltern in Paarbeziehungen bezeichnet) und 243 alleinerziehende Eltern mit Kindern zwischen 6 bis 17 Jahren zu ihren Erfahrungen mit der Beschulung ihrer Kinder zu Hause befragt.^5^ Davon waren *N* = 488 Männer und *N* = 1020 Frauen. Von den befragten Personen lebten 18 % zum Zeitpunkt des Interviews in Ostdeutschland. Eine detaillierte Beschreibung der SOEP-CoV Studie und ihres Designs findet sich in Kühne et al. (2020).

### Instrumente

Das Merkmal, das wir in unseren Analysen untersuchen, ist die subjektive Belastung der Eltern durch die Beschulung ihrer Kinder Zuhause. Das hierfür genutzte Item der SOV-CoV Befragung lautet: „Wie ist Ihre persönliche Einschätzung zu folgender Aussage? Dafür zu sorgen, dass das Kind den Schularbeiten nachkommt, wird mich überfordern.“ Das Item wurde auf einer 5‑er Skala mit Wert 1 (Stimme überhaupt nicht zu) bis Wert 5 (Stimme voll zu) abgefragt. Hierbei wurden die Eltern gebeten auf das jeweils jüngste Schulkind im Haushalt Bezug zu nehmen.

Das untersuchte Item ist so formuliert, dass es eine antizipierte Belastung erfragt und keine tatsächliche.^6^ Dennoch eröffnet die zeitliche Verortung der Befragung (nämlich direkt während des ersten Lockdowns im Frühjahr 2020) und die Bitte an die Befragungspersonen, Auskunft zu ihren Erfahrungen mit den Corona-bedingten politischen Maßnahmen zu geben, die Möglichkeit das Items auch zur Untersuchung der tatsächlichen subjektiven Belastung der Eltern zu nutzen. Um quantifizieren zu können wie gut dies gelingt, untersuchen wir, ob das Items in vergleichbaren Stichproben und zu ähnlichen Zeitpunkten das Gleiche misst. Hierzu machen wir uns das Design der SOEP-CoV Studie zu Nutze, indem wir das subjektiven Belastungsempfinden, das in der Tranche 1 gemessen wurde, mit dem vergleichen, das in Tranche 2 ermittelt wurde. Ebenso verfahren wir mit Tranche 2 und Tranche 3 sowie mit Tranche 3 und Tranche 4. Zum Vergleich nutzen wir einen gewichteten t‑Test.^7^ Insgesamt berechnen wir drei t‑Tests mit den tranchenspezifischen Stichprobengrößen N_Tranche1_ = 455, N_Tranche2_ = 583, N_Tranche3_ = 273 und N_Tranche4_ = 197 sowie den zugehörigen gewichteten Mittelwerten von M_Tranche1_ = 2,16 (SD = 1,29), M_Tranche2_ = 2,28 (SD = 1,22), M_Tranche3_ = 2,36 (SD = 1,35), M_Tranche4_ = 2,33 (SD = 1,16). Im Ergebnis deutet keiner dieser Tests auf signifikante Mittelwertsunterschiede im Belastungsempfinden zwischen aufeinanderfolgenden Tranchen hin (d. h. die zugehörigen *p*-Werte sind in jedem Fall wesentlich größer als 0,05). Wir untersuchen die subjektiv wahrgenommene Belastung der Eltern in Bezug auf die verschiedenen Haushaltskontexte und sozio-ökonomischen Faktoren, die wir in unseren theoretischen Überlegungen als möglicherweise erklärungsträchtig ermittelt haben. Insbesondere unterscheiden wir in unseren Betrachtungen alleinerziehende Eltern und Eltern in Paarbeziehungen. Die entsprechende Differenzierung erfolgte auf Basis der Haushaltstyp-Variable des SOEP, die Kategorien für Alleinerziehende und Eltern mit Kindern im Haushalt ausweist.

Des Weiteren differenzieren wir in unseren Analysen Eltern nach ihrem Bildungsabschluss. Konkret wurde der elterliche Bildungsabschluss durch den Bildungsabschluss der Befragungsperson operationalisiert. Insgesamt unterscheiden wir drei Gruppen von elterlichen Bildungsniveaus: hoch, mittel und niedrig. Für diese Unterscheidung nutzen wir die CASMIN Klassifikation in der folgenden Art: niedriges Bildungsniveau entspricht den CASMIN Klassen 0,1a, 1b, 2b, ein mittleres Bildungsniveau den CASMIN Klassen 1c, 2a, 2c und ein hohes Bildungsniveau den CASMIN Klassen 3a, 3b.

Außerdem beziehen wir den Erwerbstatus des befragten Elternteils zu Zeiten des Corona-bedingten Lockdowns in unsere Analysen ein. Diese Information wurde ebenfalls in der SOEP-CoV Befragung erhoben und entspricht somit dem Erwerbsstatus zum Befragungszeitpunkt. Wir unterscheiden zwischen Vollzeit-Erwerbstätigen, Eltern, die in Teilzeit arbeiten, Eltern, die keinerlei Erwerbstätigkeit nachgehen und Eltern, die einer anderen als den genannten Erwerbsformen nachgehen, z. B. in Ausbildung oder in Kurzarbeit sind.

Zudem kontrollieren wir für das Geschlecht der Befragungsperson und ihren Migrationshintergrund. Hierbei operationalisieren wir eine Person als Person mit Migrationshintergrund, wenn sie selbst oder mindestens eines ihrer Elternteile nach Deutschland migriert ist. Auch eine regionale Verortung der befragten Haushalte in Form einer Unterscheidung zwischen Ost- und Westdeutschland fließt in die Analyse ein.

Als Kontextvariablen fließen zudem die Anzahl von Kindern zwischen 1 und 17 Jahren^8^, die zum Zeitpunkt der Befragung im Haushalt lebten, in die Analyse ein sowie das Alter des jüngsten Schulkindes, auf das die Frage nach dem Belastungsempfinden durch die Anforderungen aufgrund der Schulschließungen abzielt. Außerdem kontrollieren wir in unserer Analyse für die durchschnittliche Zeit, die im Jahr 2019 werktags für die Kinderbetreuung von der befragten Person aufgebracht wurde.

Als einen möglicherweise unterstützenden Faktor von Seiten der Schule (auf die das jüngste Schulkind geht und auf welches sich wiederum die Belastungsfrage bezieht) betrachten wir, ob die Schule für die Zeit der Schulschließungen Lernmaterial auf mehreren der folgenden Wege zur Verfügung gestellt hat: per E‑Mail, auf einem Server und/oder einer Cloud, per Videokonferenzschaltung, vor Schulschließung, überhaupt nicht oder auf einem anderen Weg (z. B. persönlich durch die Lehrkraft).

Tab. 1 zeigt eine Zusammenfassung der in dieser Analyse genutzten Variablen inklusive gewichteten Mittelwerten bzw. Anteilen. Kategorien mit weniger als 30 Beobachtungen sind eingeklammert. Diesen Werten wohnt eine große Unsicherheit in Bezug auf die Grundgesamtheit der zugehörigen Elterngruppen inne. Ergebnisse, die sich aus Auszählungen bzw. Analysen in diesen Kategorien ergeben, werden im Nachfolgenden von jeglicher Interpretation ausgeschlossen. Insbesondere die Kategorie der alleinerziehenden Männer ist im Vergleich zur Kategorie der alleinerziehenden Frauen anteilsmäßig viel zu groß. Die Herleitung der zur Gewichtung genutzten Faktoren ist im folgenden Abschnitt „Methoden- und Analysemodell“ beschrieben.

**Table 1:** 

	Eltern in Paarbeziehungen	Alleinerziehende Eltern	Total
*Subjektive Belastung*	2,24	2,57	2,26
*Bildungsniveau*			
Hoch	0,30	0,17	0,29
Mittel	0,53	0,49	0,52
Niedrig	0,17	(0,33)	0,19
*Erwerbsstatus In 2020*			
Vollzeit	0,49	0,46	0,48
Teilzeit	0,25	0,24	0,25
Nicht erwerbstätig	0,15	0,20	0,16
Anderer	0,13	(0,10)	0,12
*Geschlecht Elternteil*			
Männlich	0,48	(0,23)^a^	0,45
Weiblich	0,52	0,77	0,55
*Migrationshintergrund*			
Ja	0,39	0,34	0,37
Nein	0,61	0,66	0,63
*Region*			
Ostdeutschland	0,15	0,26	0,17
Westdeutschland	0,85	0,74	0,83
*Anzahl Kinder im Haushalt*			
1	0,39	0,58	0,41
2 und mehr	0,61	0,42	0,59
*Alter des jüngsten Kindes*			
Unter 10 Jahre	0,29	(0,21)	0,28
11 bis 14 Jahre	0,39	0,38	0,38
Älter als 14 Jahre	0,33	0,41	0,34
*Kinderbetreuungszeit in 2019 werktags*			
0 bis 3 h	0,66	0,60	0,66
4 bis 7 h	0,24	0,32	0,24
Mehr als 7 h	0,10	(0,08)	0,09
*Schulmaterial über mehrere Wege erhalten*			
Nein	0,63	0,56	0,62
Ja	0,37	0,44	0,38
Stichprobengröße (ungewichtet)	1244	243	1508^b^

*Anmerkung*: Gewichtete Angaben. Kategorien mit weniger als *N* = 30 Fällen sind eingeklammert

^a^ Im Vergleich zur Grundgesamtheit ist dieser Wert zu hoch. Schätzungsweise gibt es circa 7 bis 8 % alleinerziehende Väter in Deutschland

^b^ Der Familienstatus von *N* = 21 dieser *N* = 1508 Personen ist unbekannt

### Methode und Analysemodell

Die Stichprobe der SOEP-CoV Befragung ist (ungewichtet) im Vergleich zur Grundgesamtheit aller Eltern mit Kindern zwischen 6 und 17 Jahren, die eine Schule besuchen, verzerrt, zum Beispiel bezüglich des Geschlechts des Elternteils. Daher wurden – um Aussagen über die Grundgesamtheit machen zu können – alle Analysen gewichtet durchgeführt. Die entsprechenden Gewichtungsfaktoren ergeben sich durch einen mehrstufigen Prozess, der vom Haushaltsgewicht der SOEP Stichproben 2019 ausgeht und bei dem in einem letzten Schritt ein Gewichtungsfaktor auf Personenebene für die CATI Befragung der SOEP-CoV Studie hergeleitet wird. Die Gewichtungsfaktoren der SOEP-CoV Studie sind in Bezug auf eine Vielzahl von Faktoren auf Haushalts- und Personenebene *unit nonresponse* adjustiert (u. a. bzgl. Erwerbsstatus im Haushalt und individuell, persönliches und Haushaltseinkommen, Anzahl Personen im Haushalt, Haushaltstyp, Bildungsniveau der Haushaltsmitglieder, Migrationshintergrund, ob eine Person (im Haushalt) in einem system-relevanten Beruf arbeitet, die Covid-19 Infektionsrate auf Kreisebene zum Zeitpunkt des Interviews) und an die Grundgesamtheit der Privathaushalte und deren Haushaltsmitglieder an entsprechende Populationsverteilungen (u. a. Alter, Geschlecht, Haushaltsgröße, Staatsbürgerschaft, Gemeindegröße und Bundesland), die dem Mikrozensus 2018 entnommen wurden, angepasst.^9^ Eine detaillierte Beschreibung des Teilnahmeverhaltens der SOEP Haushalte an der der SOEP-CoV Studie und des zugehörigen Gewichtungsverfahrens findet sich in Siegers et al. (2020).

Insgesamt gibt es nur für rund 70 % der befragten Eltern vollständig beobachtete Werte. Der zugrundeliegende *item nonresponse* Mechanismus ist nachweislich^10^ nicht vollständig zufällig. Aus diesem Grund ersetzen wir fehlende Werte mittels multipler Imputation. Konkret nutzen wir *multivariate imputation by chained equations* („mice“) mit *Bayesian polytomous regression* („polyreg“) für kategoriale und *classification and regression trees *(„cart“) für stetige Variablen mit fehlenden Werten.^11^ Wie von Kim et al. (2006) vorgeschlagen, fließt das *unit nonresponse*-adjustierte und randangepasste SOEP-CoV Surveygewicht als erklärende Variable in die zugehörigen Imputationsmodelle ein. Insgesamt wurden 20 imputierte Datensätze erzeugt.

In einem ersten Schritt haben wir die durch die Schulschließungen empfundene Belastung der Eltern getrennt nach Erziehendenstatus (d. h. alleinerziehend oder nicht) mittels gewichteten Populationsmittelwerten geschätzt. Diese Auswertung haben wir in einem zweiten Schritt verfeinert und uns die gewichteten Populationsschätzer mit Blick auf das elterliche Bildungsniveau angeschaut. Hierbei haben unter Verwendung der CASMIN Klassifikation zwischen niedrig, mittel und hoch gebildeten Eltern unterschieden. Ein t‑Test zeigte uns dann an, ob es statistisch signifikante Unterschiede (auf einem Signifikanzniveau von 5 %) zwischen den untersuchten Bildungsgruppen bei Alleinerziehenden und Eltern in Paarbeziehungen gibt. In den Variablen, die für diese Analysen genutzt wurden, lag der Anteil an durch *item nonresponse* erzeugten fehlenden Werten unter 5 %. Daher wurden die entsprechenden Analysen nicht auf imputierten Daten durchgeführt. In einem dritten Schritt gab eine multivariate Analyse darüber Aufschluss, inwieweit die als theoretisch relevant identifizierten Faktoren das Belastungsempfinden der Eltern (durch die zusätzlichen Anforderungen durch die Schulschließungen) erklären und für welche Gruppen es signifikante Unterschiede hinsichtlich des Belastungsempfindens gibt. Die zugehörige linear Regression wurde auf den zuvor imputierten Daten berechnet.^12^

## Ergebnisse

### Deskriptive Befunde

**Figure 1:**
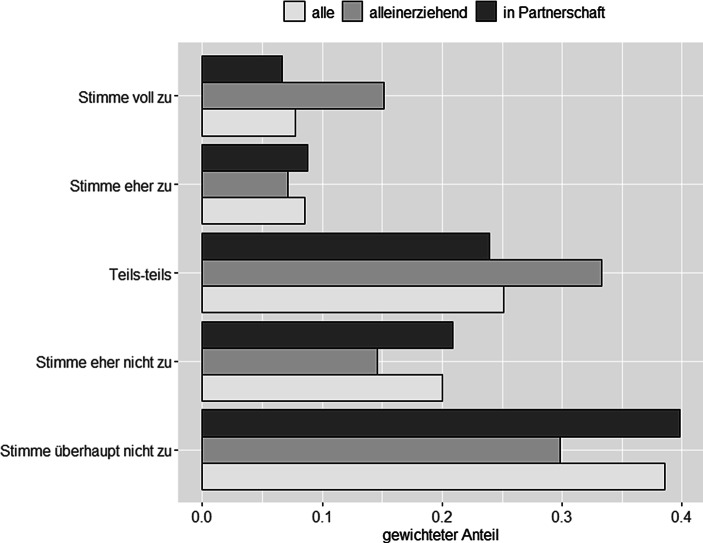

Abb. 1 zeigt die (gewichtete) Verteilung der Skalenwerte, die Eltern bei der Frage nach ihrem subjektiven Belastungsempfinden angegeben haben. Wir sehen, dass über ein Drittel aller Eltern den Skalenwert („Stimme überhaupt nicht zu“) angegeben haben, und dies unabhängig davon ob sie alleinerziehend sind oder nicht. Dennoch gaben im Vergleich zu Eltern in Paarbeziehungen, Alleinerziehende weniger häufiger an, sich durch die Schulschließungen überhaupt nicht belastet zu fühlen (40 % vs. 30 %). Auffällig ist die große Häufung von Alleinerziehenden, die sich übermäßig belastet fühlten (Skalenwert 5 „Stimme voll zu“). Im Vergleich zu 7 % aller Eltern in Paarbeziehungen, haben 15 % aller alleinerziehenden Eltern diese Skalen-Ausprägung gewählt. Generell finden wir allerdings auch eine große Tendenz aller Eltern anzugeben, dass sie sich „teils/teils“ belastet bzw. überfordert fühlten, wobei auch hier der Anteil von Alleinerziehenden wieder größer ist als der Anteil von Eltern in Paarbeziehungen. Diese tendierten eher dazu sich dem Bereich weniger stark belastet bzw. überfordert zuzuordnen.

Dies deutet darauf hin, dass sich alleinerziehende Eltern in der Tat sehr stark von den Belastungen, die durch die Schulschließungen auf sie zukamen, belastet wenn nicht gar überfordert sahen. Ein Blick auf den gewichteten Populationsmittelwert (Tab. 2) stützt diese Vermutung. Hier finden wir einen signifikanten Unterschied im Belastungsempfinden von Alleinerziehenden im Vergleich zu Eltern in Paarbeziehungen. (Mittelwertsunterschiede wurden mittels t‑Test untersucht. Als signifikante Unterschiede bezeichnen wir solche mit einem *p*-Wert kleiner als 0,05.) Somit stützen unsere Auswertungen auf Basis der SOEP-CoV Daten unsere Hypothese 1, dass sich Alleinerziehende stärker durch die Anforderungen belastet sahen, denen sie sich durch den akuten Stressor „Schulschließung“ ausgesetzt sahen, als Eltern in Paarbeziehungen.

**Table 2:** 

	Mittelwert (gewichtet)	Standardabweichung (gewichtet)	Stichprobengröße
Alle Elternteile	2,27	1,27	1492 ^a^
Elternteile In Partnerschaft	2,22	1,24	1233
Alleinerziehende Elternteile	2,63	1,37	243

Anmerkung: Gewichtete Auswertung

^a^ *N* = 16 Eltern haben keine Angaben zu ihrem Belastungsempfinden gemacht. Davon waren *N* = 5 alleinerziehend

**Figure 2:**
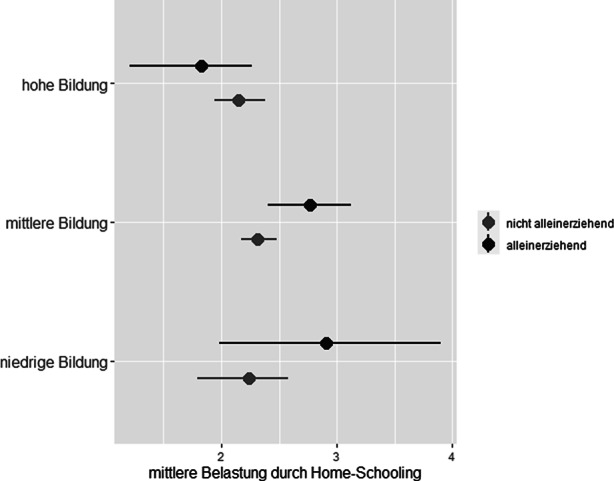

Mit Blick auf das Bildungsniveau finden wir, dass unabhängig von der Familienform sich Eltern mit einem niedrigeren Bildungsniveau stärker belastet fühlten als Eltern mit einem hohen Bildungsniveau, siehe Abb. 2 und Tab. 3. Die Abweichungen von den zugehörigen Gruppenmittelwerten sind sowohl bei Eltern in Paarbeziehungen als auch bei Alleinerziehenden signifikant. Bei Eltern in Paarbeziehungen trifft dies allerdings nur für diejenigen mit einem niedrigen Bildungsniveau im Vergleich zu denjenigen mit einem hohen zu, d. h. es kann kein signifikanter Unterschied zwischen Eltern mit einem hohen und einem mittleren Bildungsniveau ausgemacht werden. Gravierender sind die Unterschiede bei alleinerziehenden Eltern. Hier unterschieden sich die Abweichungen vom Gruppenmittelwert für alle drei Ausprägungen des Bildungsniveaus signifikant voneinander. Wir sehen darin ein Indiz zur Bestätigung unserer zweiten Hypothese, dass das elterliche Bildungsniveau als familiäre Ressource kompensatorisch auf das elterliche Belastungsempfinden durch die Beschulung der Kinder zu Hause wirkt. Allerdings zeigt der empirische Befund auch, dass der Zusammenhang zwischen dem elterlichen Bildungshintergrund und dem elterlichen Belastungsempfinden für alleinerziehende Eltern stärker ist als für Eltern in Paarbeziehungen.

**Table 3:** 

	Eltern in Paarbeziehungen		Alleinerziehend	
	Gruppenmittelwert	Abweichung vom Gesamtgruppenmittelwert ^a^	Gruppenmittelwert	Abweichung vom Gesamtgruppenmittelwert ^a^
Niedrige Bildung	2,15	−0,07	2,91	0,28
Mittlere Bildung	2,31	0,09	2,77	0,14
Hohe Bildung	2,14	−0,08	1,83	−0,80

*Anmerkung:* Gewichtete Auszählung.

^a^ Die entsprechenden Gesamtgruppenmittelwerte *m*_*allein*_ = 2,63 und *m*_*paar*_ = 2,22 für Alleinerziehende bzw. Eltern in Paarbeziehungen finden sich in Tab. 2

### Befunde aus der multivariaten Regression

Zur Untersuchung der Hypothesen 2 bis 5, die auf das Belastungsempfinden Alleinerziehender nach ihren unterschiedlichen Situationen und häuslichen Kontexten abzielen, nutzen wir eine multivariate Analyse. Konkret schätzen wir ein lineares Regressionsmodell auf den zuvor imputierten Datensätzen und poolen die Ergebnisse im Nachgang. Die Variablen, die uns hierbei interessieren sind das elterliche Bildungsniveau (Hypothese 2), das Alter des Schulkinds (Hypothese 3) und die Erwerbssituation zu Zeiten der Schulschließungen (Hypothese 4). Wir kontrollieren in unserer Analyse für das Geschlecht und den Migrationshintergrund des Elternteils, die Anzahl der Kinder im Haushalt, die regionale Verortung des Haushalts (Ost- oder Westdeutschland) sowie für die Betreuungszeit, die das alleinerziehende Elternteil 2019 für die Kinderbetreuung aufgebracht hat. Außerdem untersuchen wir die Wirkung der Übermittlung von Beschulungsmaterial über mehrere Wege (E-Mail, Server/Cloud, Konferenzschaltung, vor Schulschließung, anderes) (Hypothese 5). Tab. 4 zeigt die Ergebnisse der multivariaten Analyse. Die Ergebnisse einer vergleichbaren multivariaten Analyse hinsichtlich des Belastungsempfindens von Eltern in Paarbeziehungen finden sich im Anhang in Tab. 5.

**Table 4:** 

Variable	Referenzkategorie	Koeffizient
*Intercept*		2,10*
*Bildungsniveau*	Hoch (CASMIN 3a, 3b)	
Mittel (CASMIN 1c, 2a, 2c)		0,77*
Niedrig (CASMIN 0, 1a, 1b, 2b)		(1,09*)
*Alter des jüngsten Schulkinds*	Unter 10 Jahre ^a^	
11 bis 14 Jahre		−0,38
Älter als 14 Jahre		−0,53
*Erwerbsstatus in 2020*	Vollzeit	
Teilzeit		0,19
Nicht erwerbstätig		−0,71*
Anderes		(−0,78*)
*Geschlecht Elternteil*	Männlich ^a^	
Weiblich		0,28
*Anzahl Kinder im Haushalt*	1	
2 und mehr		−0,28
*Migrationshintergrund*	Nein	
Ja (direkt oder indirekt)		0,36
*Region*	Ostdeutschland	
Westdeutschland		0,29
*Betreuungszeit 2019 werktags*	0–3 h	
4 bis 7 h		0,19
Mehr als 7 h		−0,27
*Schulmaterial über mehrere Wege*	Nein	
Ja		−0,42*
Stichprobengröße		243
R^2^ (adj.)		0,21

*Anmerkungen*: Gewichtete lineare Regression auf 20 imputierten Datensätzen. Abhängige Variable: Belastung durch Schulschließung („Dafür zu sorgen, dass das Kind den Schularbeiten nachkommt, wird mich überfordern.“) gemessen auf Skala 1 (Stimme überhaupt nicht zu) bis 5 (Stimme voll zu). Kategorien mit weniger als 30 Beobachtungen sind eingeklammert

*p < 0.05

^a^ Insgesamt nur *N* = 32 alleinerziehende Männer und *N* = 28 alleinerziehende Eltern mit einem jüngsten Schulkind unter 10 Jahren. Eine alternative Modellierung ohne alleinerziehende Männer gibt ähnliche Ergebnisse

Wir sehen, dass auch unter Kontrolle möglicherweise relevanter individueller und häuslicher Merkmale der starke Effekt des Bildungsniveaus auf das elterliche Belastungsempfinden in Bezug auf die Beschulung ihrer Kinder zu Hause bestehen bleibt. So fühlen sich alleinerziehende Eltern, die kein hohes Bildungsniveau haben, auch unter gleichen häuslichen oder individuellen Gegebenheiten deutlich mehr belastet als solche mit einem hohen Bildungsniveau. Dies unterstützt unsere zweite Hypothese und unterstreicht die starke kompensatorische Wirkung des Bildungsniveaus auf das Belastungsempfinden bei Alleinerziehenden hinsichtlich des Stressors „Schulschließung“. Wir finden indes kein Indiz, dass mit dem zunehmenden Alter des jüngsten Schulkinds das Belastungsempfinden alleinerziehender Eltern abnimmt. Somit können wir unsere Hypothese, dass das Belastungsempfinden Alleinerziehender mit zunehmendem Alter des Kindes durch einen geringeren Betreuungsaufwand im Vergleich zu den größeren Herausforderungen, die mit der Bearbeitung schulischer Inhalte einhergehen, relativ gesehen abnimmt (d. h. Hypothese 3), auf Basis unserer Auswertungen mit den SOEP-CoV Daten nicht bestätigen. Hinsichtlich der Erwerbssituation ergibt die Analyse, dass sich nicht erwerbstätige, alleinerziehende Eltern deutlich weniger durch die Anforderungen der Beschulung Zuhause belastet sehen als solche die in Vollzeit oder Teilzeit arbeiten. Das bestätigt unsere vierte Hypothese.

In einem letzten Schritt adressieren wir den möglichen Einfluss der Art der Übermittlung von Beschulungsmaterial an die alleinerziehenden Eltern. Hier konnten wir einen signifikanten Einfluss in Höhe von -0,42 ausmachen. Das bedeutet, dass wenn alleinerziehenden Eltern Beschulungsmaterial auf mehreren Wegen zugestellt wurde, hat dies ihr Belastungsempfinden wesentlich vermindert. Somit ist eine verstärkte Unterstützung durch die Schule ein stresshemmender Faktor für alleinerziehende Eltern. Dies unterstützt die Richtigkeit unserer 5. Hypothese.

Mit Blick auf die Kontrollvariablen finden wir hinsichtlich des elterlichen Geschlechtes keinerlei Anhaltspunkte, dass sich alleinerziehende Väter im Vergleich zu alleinerziehenden Müttern stärker belastet fühlen. Für eine solche Untersuchung ist die gewählte Datenbasis mit *N* = 29 alleinerziehenden Männern aber einfach nicht effektiv genug. Wir finden auch keine Unterschiede zwischen alleinerziehenden Eltern mit und ohne Migrationshintergrund. Gleiches gilt für die regionale Verortung des Alleinerziehendenhaushalts: Es können keine signifikanten Unterschiede im Belastungsempfinden zwischen ost- und westdeutschen Alleinerziehenden ausgemacht werden. Die Betreuungszeit im Vorjahr zeigt auch keinen Einfluss auf das Belastungsempfinden. Ein Teil eines möglichen Effekts, der hier zu vermuten steht, wird vermutlich von der Erwerbssituation des Alleinerziehenden abgefangen. Hinweise darauf liefert ein Korrelationswert von 0,13 (*polychoric correlation*) zwischen beiden Variablen.

Generell ist in Bezug auf die durchgeführte Analyse anzumerken, dass sie mit einem (adjustierten) R^2^ von 0,21 eine sehr gute Modellgüte aufweist, d. h. das betrachtete Modell erklärt 21 % der Variabilität in den Daten zum Belastungsempfinden von Alleinerziehenden. Das weist mit Blick auf die Stichprobengröße von *N* = 243 alleinerziehenden Eltern auf eine hohe Bedeutung der gefundenen Effekte hin.

Eine vergleichbare multivariate Analyse bezogen auf Eltern in Paarbeziehungen zeigt (siehe Tab. 5), dass der elterliche Bildungshintergrund unter Kontrolle der unter Abschn. 2.2. benannten familiären und kontextuellen Merkmale keinen signifikanten Effekt auf das subjektive Belastungsempfinden der Eltern hinsichtlich der Beschulung ihrer Kinder zu Hause hat. Hier finden sich nur signifikante Zusammenhänge hinsichtlich Erwerbstätigkeit und in 2019 aufgewandter Betreuungszeit an Werktagen. Selbst die Übermittlung von Beschulungsmaterial steht nicht in einem wesentlichen Zusammenhang mit dem elterlichen Belastungsempfinden. Der Erklärungskraft des Modells für Eltern in Paarbeziehungen ist indes mit 3 % sehr gering. Das heißt, bezogen auf die betrachteten Variablen zeigen die untersuchten Eltern eine gleichartige Belastungsempfindung. Dies stützt unsere anfangs geäußerte These, dass die Schulschließungen im Frühjahr 2020 – zumindest von Eltern in Paarbeziehungen – als ein kollektives Ereignis wahrgenommen wurden, das alle gleichermaßen betrifft. Familiäre Ressourcen spielen bei der Belastungsempfinden keine oder allenfalls eine nur untergeordnete Rolle.

Mit Blick auf den theoretischen Rahmen des FSM als mögliches Erklärungsmodell für das elterliche Belastungsempfinden durch die Beschulung ihrer Kinder zu Hause lässt sich somit sagen, dass die familiäre Ressource „Bildungskapital“ bei Alleinerziehenden eine wesentliche Rolle hinsichtlich des elterlichen Stresses zu spielen scheint, im Gegensatz zu Eltern in Paarbeziehungen. Der Erwerbsstatus und damit vermutlich die zur Beschulung der Kinder verfügbare Zeit hat indes in beiden Familienformen einen merkbaren Einfluss auf das subjektive Belastungsempfinden. So fühlten sich Eltern, die im Lockdown im Frühjahr 2020 nicht erwerbstätig waren oder einer anderen Erwerbstätigkeit nachgingen (z. B. geringfügig beschäftig oder in Kurzarbeit waren) weniger stark belastet.

## Diskussion und Zusammenfassung

In diesem Beitrag haben wir uns mit der Frage beschäftigt, wie sich die Corona-bedingten Schulschließungen im April und Mai 2020 auf das Belastungsempfinden der Eltern auswirkten. Konkret ging es darum zu untersuchen, ob sich Eltern aus verschiedenen sozialen Gruppen und in unterschiedlichen familiären Kontexten sowie Erwerbsituationen den Anforderungen, die durch die Schulschließungen auf sie zukamen, in gleichem Maße gewachsen sahen. Insgesamt mussten alle Eltern von Schulkindern Lösungen finden, mit der neuen Situation und vor allem den neuen Anforderungen umzugehen. Für unsere Betrachtungen haben wir den theoretischen Rahmen eines erweiterten Family Stress Models (FSM) herangezogen, in dem die Schulschließungen einen akuten Stressor auf das elterliche Belastungsempfinden darstellen und familiäre, immaterielle Ressourcen wie der formale Bildungsabschluss und die Erwerbssituation der Eltern stresshemmende bzw. -steigernde Faktoren darstellen. Aus der Perspektive des Vulnerabilitäts-Stress-Adaptations-Modells fassten wir diese Ressourcenunterschiede und insbesondere die mit der Familienform Alleinerziehend einhergehenden Einschränkungen als Vulnerabilität dieser Familienform auf, die sich besonderen Anpassungsnotwendigkeiten ausgesetzt sieht. Da die Zustimmung zu den Schulschließungen in der Bevölkerung im März 2020 generell groß war, ist davon auszugehen, dass die meisten Eltern bereit waren Ressourcen für die Beschulung ihrer Kinder zu Hause aufzubringen. Dies zeigt sich auch in unseren Analysen mit den SOEP-CoV Daten, die den Zeitraum der Schulschließungen umfassen. Im Durchschnitt berichteten die befragten Eltern eine eher mittlere Belastung bzw. erwartete Überforderung durch die Anforderungen, die durch die Schulschließungen auf sie zukamen und zukommen sollten. Dabei beobachteten wir jedoch auch (signifikante) Unterschiede zwischen verschiedenen Elterngruppen. Eltern mit einem niedrigeren Bildungsabschluss fühlten sich stärker belastet bzw. überfordert als Eltern mit einem hohen Bildungsabschluss. Dies mag der Tatsache geschuldet sein, dass Eltern mit einem niedrigen Schulabschluss im Allgemeinen eine geringere Nähe zur Schule und zum Schulstoff haben als Eltern mit einem hohen Bildungsabschluss. Insbesondere alleinerziehende Eltern berichteten von einer sehr starken Belastung durch die Beschulung Zuhause, d. h. bei Ihnen steht der Stressor „Schulschließung“ in besonders starkem Zusammenhang mit dem Belastungsempfinden. Das liegt sicherlich daran, dass sich gerade diese Gruppe gezwungen sah, die neuen Anforderungen alleine abzuleisten. Hinzu kommt, dass es alleinerziehenden Eltern weit weniger häufig möglich war, im Home-Office zu arbeiten als Eltern in Paarbeziehungen. Hier finden sich z. B. in den SOEP-CoV Daten für den Zeitraum April/Mai 2020 Werte von 33 % für Alleinerziehende, im Vergleich zu 38 % Eltern in Paarbeziehungen. Zu Beginn der Zeit der Schulschließungen standen freilich die besonderen Herausforderungen und Belastungen, welche die Corona-Pandemie und die damit verbundenen Regelungen und Maßnahmen für Alleinerziehende bedeuteten nicht im Zentrum der politischen Aufmerksamkeit. Erst im Verlauf des Aprils rückte diese Elterngruppe verstärkter in den Blick der Regierungen und es kam beispielsweise zu einer Erweiterung des Anspruchs auf Notbetreuung für deren Kinder.^13^ Demensprechend gab es für sie, ebenso wie für Eltern, die in einem sogenannten system-relevanten Beruf arbeiteten, im Verlauf des Aprils und Mais, zunehmend die Möglichkeit ihre Kinder in eine Notbetreuung zu geben.^14^ Jedoch dürfte diese Option Alleinerziehende nur zu einem geringen Teil bei den Beschulungsaufgaben entlastet haben. Die Notbetreuung in Schulen war im Allgemeinen über alle Jahrgangstufen hinweg organisiert. Somit dürfte es für die betreuende Person schwierig gewesen sein, auf die individuellen Lernbedürfnisse eines Kindes einzugehen. Wir konnten in diesem Zusammenhang zudem zeigen, dass sich die empfundene Belastung durch die Schulschließungen nur bei denjenigen Alleinerziehenden reduzierte, die zum Zeitpunkt der Schulschließungen nicht erwerbstätig waren, während sich bei einer Teilzeit- im Vergleich zu einer Vollzeiterwerbstätigkeit kein solcher Reduktionseffekt nachweisen lässt. Als eine effektive Unterstützung durch die Schule (mit Blick auf die empfundene Belastung durch eine Beschulung Zuhause) betrachteten alleinerziehende Eltern die Bereitstellung von Lernmaterialien auf verschiedenen Wegen (z. B. per E‑Mail, über eine Cloud und/oder direkt über die Lehrkraft). Dies unterstreicht, wie wichtig institutionelle Unterstützung (z. B. durch die Schule) für Alleinerziehende bei der Beschulung und Betreuung ist. Generell lässt sich sagen, dass sich das FSM zur Beschreibung der Wirkung der immateriellen, familiären Ressourcenausstattung auf die subjektive Belastungsempfinden von Alleinerziehenden eignet. Dies gilt aber nicht für Eltern in Paarbeziehungen. Die Erklärung hierfür ist die breite Zustimmung der Eltern zu den Schulschließungen im Frühjahr 2020. Eine solche Haltung nivelliert die Wirkung von Schulschließungen als akutem Stressor auf elterliche, negative Emotionen. Fehlt jedoch eine breite Zustimmung in der Bevölkerung zu Schulschließungen (wie dies eventuell im zweiten Corona-bedingtem Lockdown am Jahresende 2020 und Jahresbeginn 2021 der Fall sein könnte) könnte auch das subjektive Belastungsempfinden in anderen Gruppen, die weniger durch auf Dauer gestellte Vulnerabilitäten gekennzeichnet sind, steigen.

In welchem Maße die mit den Schulschließungen einhergehenden unterschiedlichen Belastungssituationen sich auch in Unterschieden in der tatsächlichen schulbezogenen Begleitung der Kinder durch ihre Eltern niedergeschlagen hat und welche mittel- und langfristigen Effekte dies auf deren weitere Bildungsentwicklung haben wird, kann hier und jetzt noch nicht beantwortet werden. Das Eintreten derartiger Effekte hinsichtlich einer Erhöhung der sozialen (Bildungs‑)Ungleichheit ist mit Blick auf die bereits angeführten Studien etwa zu Ferieneffekten zu erwarten (vgl. Siewert 2013 und Cooper et al. 1996). Längsschnittstudien wie das SOEP oder auch das Nationale Bildungspanel (NEPS) eröffnen in dieser Hinsicht einiges an zukünftigen Analysemöglichkeiten.

Jedoch muss an dieser Stelle auch betont werden, dass Ferienstudien über Situationen berichten, die kaum mit der gleichzeitigen Betroffenheit von Eltern und Kindern durch die deutschlandweiten Schulschließungen zu vergleichen sind. Schulferien sind Bestandteil einer auf Dauer gestellten, wiederkehrenden und letztlich *normalen* Situation, die sich von dem hier fokussierten disruptiven Ereignis mit kollektivem Bezug unterscheidet. Entsprechend lässt sich aktuell auch nicht abschließend beurteilen, inwiefern die Verarbeitungsmuster und -strategien unterschiedlicher Elterngruppen in derartigen Situationen weitere Anpassungen vorhandener theoretischer Modelle (wie dem FSM) notwendig machen. Dabei ist vor allem zu beachten, dass Ereignisse mit kollektivem Bezug, wie etwa auch Lowe et al. (2012) zeigen konnten, zu einer Veränderung in den Rahmenbedingungen führen können, was etwa die Vulnerabilität der Familie reduzieren kann. Um Verknüpfungen zu den in diesem Feld ebenfalls wichtigen Diskussionen über die Relevanz familiärer Resilienz (Walsh 2016) als Schutz- und Bewältigungsfaktor herzustellen, wäre es jedoch notwendig, die Familien insgesamt in den Blick zu nehmen, was im Rahmen der hier vorgestellten Studie so nicht möglich war. Die besondere Relevanz der hier berichteten Befunde liegt jedoch auch in der belastbaren Aussagekraft der Daten im Gegensatz zu anderen aktuellen Studien, die sich mit Zusammenhängen zwischen der Pandemiesituation und familialen Verarbeitungsmechanismen auseinandersetzen (etwa Daks et al. 2020).

So stellt sich etwa – und dies kann mit den aktuell verfügbaren Daten nicht abgebildet werden – die Frage nach der Geräteausstattung der Haushalte. Dies gibt der alten Frage nach dem ersten digitalen Graben eine neue Perspektive: Können Haushalte mit Schulkindern die Gleichzeitigkeit von Home-Office-Anforderungen und der Bearbeitungserfordernisse digitaler schulischer Materialien überhaupt im Hinblick auf eine adäquate Geräteausstattung und einem stabilen Internetzugang gewährleisten? Die Antwort auf diese Frage fällt vermutlich je nach Haushaltszusammensetzung und -kontext sehr unterschiedlich aus. Generell ist dies jedoch eine Frage, der es sich mit Blick auf eine angestrebte Chancengleichheit in der Bildung lohnt nachzugehen.

Die für unsere Analysen genutzte Datenquelle weist in Bezug auf unsere Fragestellung zwei Schwachstellen auf, hinsichtlich derer es ratsam erscheint, auch andere Daten und/oder Befunde zu Rate zu ziehen. Erstens ist die Stichprobe der Alleinerziehenden mit 243 Fällen generell sehr klein, was ihre statistische Erklärungskraft bezüglich einiger relevanter Punkte schwächt. So ist z. B. die Gruppe der alleinerziehenden Männer im Vergleich zur Grundgesamtheit überproportional in der Stichprobe vertreten. Dies stellt erstmal kein Problem für unsere Fragestellung dar, da die von uns gefundenen Effekte auch bei Herausnahme der alleinerziehenden Männer aus der Stichprobe bestehen bleiben und sich somit als robust erweisen. Allerdings können mit unserer Analyse keine Aussagen hinsichtlich einer unterschiedlichen Belastung von alleinerziehenden Frauen im Vergleich zu alleinerziehenden Männern gemacht werden. Dies wäre jedoch gerade mit Blick auf die derzeit geführte Debatte zur Rolle alleinerziehender Väter (vgl. z. B. Seiffge-Krenke 2016) interessant gewesen. Darüber hinaus sind einige Indikatoren, welche in Bezug auf die Konsequenzen einer Beschulung Zuhause möglicherweise relevant sein könnten, nicht in den SOEP-CoV Daten enthalten. So lassen sich keine Befunde zur Qualität der häuslichen schulischen Betreuung durch die Eltern aus den vorhandenen Daten ableiten. Gleiches gilt für Aussagen zu den genauen innerhäuslichen Betreuungsarrangements.

Insgesamt konnten wir jedoch mit unserer Analyse aufzeigen, dass es während der Schulschließungen im April und Mai 2020 signifikante Unterschiede zwischen verschiedenen Elterngruppen hinsichtlich ihres subjektiven Belastungsempfindens mit Blick auf eine Beschulung Zuhause gab. Diese Unterschiede sind mit ziemlicher Sicherheit auch ein Ausdruck der Probleme bei der Umsetzung einer Beschulung Zuhause und somit ein Indikator dafür, wie gut Lernmaterialien abgearbeitet wurden. Dementsprechend ist davon auszugehen, dass Kinder, deren Eltern sich stark belastet fühlten, den von der Schule übermittelten Lernstoff weniger vollständig und adäquat abgearbeitet haben als Kinder, deren Eltern die Beschulung Zuhause als wenig belastend empfanden. Ob und wie mögliche Lernstaus kompensiert werden können, liegt nun insbesondere in den Händen der Schulen und Kultusministerien, die hierfür personelle Ressourcen verfügbar machen müss(t)en.

## Anhang

### Multivariate Analyse des subjektiven Belastungsempfinden der Eltern durch die Beschulung ihrer Kinder zu Hause in Paarfamilien

**Table 5:**

Variable	Referenzkategorie	Koeffizient
*Intercept*		2,18*
*Bildungsniveau*	Hoch (CASMIN 3a, 3b)	
Mittel (CASMIN 1c, 2a, 2c)		0,16
Niedrig (CASMIN 0, 1a, 1b, 2b)		0,21
*Alter des jüngsten Schulkinds*	Unter 10 Jahre ^a^	
11 bis 14 Jahre		−0,21
Älter als 14 Jahre		−0,18
*Erwerbsstatus in 2020*	Vollzeit	
Teilzeit		0,18
Nicht erwerbstätig		−0,47*
Anderes		−0,03
*Geschlecht Elternteil*	Männlich	
Weiblich		0,11
*Anzahl Kinder im Haushalt*	1	
2 und mehr		−0,01
*Migrationshintergrund*	Nein	
Ja (direkt oder indirekt)		0,07
*Region*	Ostdeutschland	
Westdeutschland		0,02
*Betreuungszeit 2019 werktags*	0–3 h	
4 bis 7 h		0,11
Mehr als 7 h		−0,33*
*Schulmaterial über mehrere Wege*	Nein	
Ja		−0,03
Stichprobengröße		1244
R^2^ (adj.)		0,03

*Anmerkungen*: Gewichtete lineare Regression auf 20 imputierten Datensätzen. Abhängige Variable: Belastung durch Schulschließung („Dafür zu sorgen, dass das Kind den Schularbeiten nachkommt, wird mich überfordern.“) gemessen auf Skala 1 (Stimme überhaupt nicht zu) bis 5 (Stimme voll zu). * *p* < 0,05. Für alle betrachteten Kategorien liegen mehr als 30 Beobachtungen vor.

## Footnotes

1 Ein Gemeinschaftsprojekt von Universität Erfurt (UE), Robert Koch-Institut (RKI), Bundeszentrale für gesundheitliche Aufklärung (BZgA), Leibniz-Zentrum für Psychologische Information und Dokumentation (ZPID), Science Media Center (SMC), Bernhard Nocht Institute for Tropical Medicine (BNITM), Yale Institute for Global Health (YIGH).

2 Zwar wurde bereits teilweise zu einem sehr frühen Zeitpunkt begonnen, krisenbezogene Daten zu sammeln, hierbei handelt es sich jedoch entweder um Befragungen, deren Stichprobendesign keine populationsbezogenen Schlüsse zulässt (so nutzt etwa die Studie des Deutschen Jugendinstituts eine Convenience-Stichprobe, vgl. Langmeyer et al. 2020) oder die Studien haben andere inhaltliche Schwerpunktsetzungen (so konzentriert sich die Mannheimer Corona Studie, die bereits seit Mitte März Daten sammelt, vor allem auf die wirtschaftlichen Auswirkungen der Pandemie auf die Haushalte, vgl. https://www.uni-mannheim.de/gip/corona-studie/).

3 Diese Menge an Haushalten gehört der sogenannten „zentralen Befragung des SOEP“ an. Sie sind mit einem hohen Risiko des kompletten Ausfalls aus dem Panel behaftet und werden daher generell nicht für Sonderbefragungen des SOEP herangezogen. Insgesamt, machen sie 8,8 % der gesamten SOEP Stichprobe aus.

4 Die entsprechende Präambel zur Motivation und Einleitung in die Studie findet sich in den Fragebögen der Studie, die unter https://www.soep-cov.de/Methodik verfügbar sind.

5 *N* = 21 Eltern konnten keinem Erziehendenstatus zugewiesen werden, da (im Moment) keine entsprechenden Daten vorliegen.

6 Der Grund für diese Inkonsistenz liegt darin, dass zum Zeitpunkt der Fragebogenerstellung ein Lockdown zwar zu erwarten, aber der Zeitpunkt der tatsächlichen Umsetzung unklar war. Die erste Tranche der SOEP-CoV Studie fiel somit eher ungeplant in die zweite Woche des Lockdowns. Um den Panelcharakter der SOEP-CoV Studie zu wahren, wurde auch nach der ersten Tranche der Fragetext des Items beibehalten.

7 Wir nutzen einen gewichteten t‑Test, um die Surveygewichte, die auch in unsere Analysen einfließen zu berücksichtigen. Auf diese Art kompensieren wir für selektiven Ausfall bei der Teilnahme an der Studie, siehe hierzu auch Abschn. 2.3. Konkret nutzen wir die statistische Software R (Version 4.0.3) und die Funktion „wtd.t.test“ aus dem R Paket „weights“ (Version 1.0.1).

8 Die entsprechenden Informationen wurden den SOEP Daten 2019 entnommen. Für 2020 liegen außer den SOEP-CoV Daten noch keine finalen Befragungsdaten vor.

9 Hierbei wird von der Annahme ausgegangen, dass sich die Population bezüglich der zur Randanpassung genutzten Populationsverteilungen nicht wesentlich zwischen 2018 und 2020 verändert hat.

10 Um dies zu zeigen, wurde ein entsprechender Test von Little (1988) genutzt, implementiert in der „LittleMCAR“ Funktion des R Pakets „BaylorEdPsych“ (vgl. https://rdrr.io/cran/BaylorEdPsych/man/LittleMCAR.html).

11 Implementiert in der Funktion „mice“ des R Pakets „mice“ (vgl. https://cran.r-project.org/web/packages/mice/index.html).

12 Ein Robustheitscheck hinsichtlich der Modellannahme der Nutzbarkeit eines linearen Regressionsmodells im Vergleich zu einem Ordered Logit Modell zeigt keine Abweichungen hinsichtlich der Interpretation der Ergebnisse.

13 Darüber hinaus wurden den besonderen Bedarfen von Alleinerziehenden auch durch die Anhebung des steuerlichen Entlastungsbetrags für Alleinerziehende für die Jahre 2020 und 2021 Rechnung getragen.

14 Die Definitionshoheit, welche Berufe als systemrelevant gelten, lag und liegt hierbei in der Kompetenz der Länder, so dass auch die Nutzbarkeit von Notbetreuungen Bundesland-spezifisch unterschiedlich war und ist. Das Bundesamt für Bevölkerungsschutz und Katastrophenhilfe veröffentlichte mit Stand 30.04.2020 eine entsprechende Übersicht über die in den Bundesländern geltenden Regelungen bzw. Hinweise auf die jeweiligen Quellen dieser Regelungen zur Notbetreuung in Schulen und Kitas (https://www.bbk.bund.de/SharedDocs/Downloads/BBK/DE/Sonstiges/Covid-19_Kritis_Schulen_Kitas.html).

## References

[CR1] van Ackern I, Endberg M, Locker-Grütjen O (2020). Chancenausgleich in der Corona-Krise: Die soziale Bildungsschere wieder schließen. DDS – Die Deutsche Schule.

[CR2] Anger C, Plünnecke A (2020). Homeschooling und Bildungsgerechtigkeit.

[CR3] Autorengruppe Bildungsberichterstattung (2020). Bildung in Deutschland 2020.

[CR4] Beck U (1986). Risikogesellschaft. Auf dem Weg in eine andere Moderne.

[CR5] BMAS (2017). Lebenslagen in Deutschland. Der fünfte Armuts- und Reichtumsbericht der Bundesregierung.

[CR6] Böhm-Kasper O (2004). Belastung und Beanspruchung. Eine Untersuchung von Schülern und Lehrern am Gymnasium.

[CR7] Böllert K, Bollweg P, Buchna J, Coelen T, Otto H-U (2020). Vereinbarkeit von Familie und Erwerbstätigkeit. Handbuch Ganztagsbildung.

[CR8] Cairney J, Boyle M, Offord DR, Racine Y (2003). Stress, social support and depression in single and married mothers. Social Psychiatry and Psychiatric Epidemiology.

[CR9] Collins R (2004). Rituals of solidarity and security in the wake of terrorist attack. Sociological Theory.

[CR10] Conger RD, Elder GH, Lorenz FO, Conger KJ, Simons RL, Whitbeck LB (1990). Linking economic hardship to marital quality and instability. Journal of Marriage and the Family.

[CR11] Cooper H, Nye B, Charlton K, Lindsay J, Greathouse S (1996). The effects of summer vacation on achievement test scores: a narrative and meta-analytic review. Review of Educational Research.

[CR12] Daks JS, Peltz JS, Rogge RD (2020). Psychological flexibility and inflexibility as sources of resiliency and risk during a pandemic: Modeling the cascade of COVID-19 stress on family systems with a contextual behavioral science lens. Journal of Contextual Behavioral Science.

[CR14] Destatis, WZB (2018). Datenreport 2018. Ein Sozialbericht für die Bundesrepublik Deutschland.

[CR15] Durkheim E (1994). Die elementaren Formen des religiösen Lebens.

[CR16] Elcheroth G, Drury J (2020). Collective resilience in times of crisis: Lessons from the literature for socially effective responses to the pandemic. British Journal of Social Psychology.

[CR17] Elder GH (1999). Children of the great depression.

[CR20] Feldhaus M (2015). Familiale Einflussfaktoren auf das elterliche Schulinteresse aus der Sicht von Grundschulkindern. Zeitschrift für Familienforschung.

[CR21] Filipp S-H, Ammanns P (2018). Kritische Lebensereignisse und Lebenskrisen.

[CR22] Harknett K, Knab J (2007). More kin, less support: multipartnered fertility and perceived support among mothers. Journal of Marriage and Family.

[CR23] Heintz-Martin VK, Langmeyer AN (2020). Economic situation, financial strain and child wellbeing in stepfamilies and single-parent families in Germany. Journal of Family and Economic Issues.

[CR24] Hoover-Dempsey KV, Walker JMT, Sandler HM, Whetsel D, Green CL, Wilkins AS, Closson K (2005). Why do parents become involved? Research findings and implications. The Elementary School Journal.

[CR25] Karney BR, Bradbury TN (1995). The longitudinal course of marital quality and stability: a review of theory, method, and research. Psychological Bulletin.

[CR26] Keim-Klärner S, Klärner A, Gamper M, Keim-Klärner S, Moor I, von der Lippe Vonneilich HN (2020). Soziale Netzwerke und die Gesundheit von Alleinerziehenden. Soziale Netzwerke und gesundheitliche Ungleichheiten.

[CR27] Kim JK, Brick MJ, Fuller WA, Kalton G (2006). On the bias of the multiple-imputation variance estimator in survey sampling. Journal of the Royal Statistical Society: Series B (Statistical Methodology).

[CR28] Kühne S, Kroh M, Liebig S, Zinn S (2020). The need for household panel surveys in times of crisis: the case of SOEP-coV. Survey Research Methods.

[CR29] Langmeyer, A., Guglhör-Rudan, A., Naab, T., Urlen, M., & Winklhofer, U. (2020). Kindsein in Zeiten von Corona. Erste Ergebnisse zum veränderten Alltag und zum Wohlbefinden von Kindern. https://www.dji.de/fileadmin/user_upload/dasdji/themen/Familie/DJI_Kindsein_Corona_Erste_Ergebnisse.pdf. Zugegriffen: 22. März 2021.

[CR30] Lareau A (2000). Home advantage. Social class and parental intervention in elementary education.

[CR31] Lareau A (2003). Unequal childhoods. Class, race and family life.

[CR32] Little RJ (1988). A test of missing completely at random for multivariate data with missing values. Journal of the American statistical Association.

[CR33] Lowe SR, Rhodes JE, Scoglio AA (2012). Changes in marital and partner relationships in the aftermath of Hurricane Katrina: An analysis with low-income women. Psychology of women quarterly.

[CR42] Maaz, K., Baumert, J., & Trautwein, U. (2010). Genese sozialer Ungleichheit im institutionellen Kontext der Schule: Wo entsteht und vergrößert sich soziale Ungleichheit?. In: Baumert J., Maaz K., Trautwein U. (Hrsg.) *Bildungsentscheidungen*. VS Verlag für Sozialwissenschaften. 10.1007/978-3-531-92216-4_2

[CR34] Marjoribanks K (2002). Family and school capital. Toward a context theory of student’s school outcomes.

[CR35] Müller K-U, Samtleben C, Schmieder J, Wrohlich K (2020). Corona-Krise erschwert Vereinbarkeit von Beruf und Familie vor allem für Mütter: Erwerbstätige Eltern sollten entlastet werden. DIW Wochenbericht.

[CR36] Peuckert R (2019). Familienformen im sozialen Wandel.

[CR37] Seiffge-Krenke I, Seiffge-Krenke I (2016). Alleinerziehende Väter–Gefährdung für die Gesundheit der Kinder oder die „besseren Mütter“?. Väter, Männer und kindliche Entwicklung.

[CR38] Siegers R, Steinhauer HW, Zinn S (2020). Gewichtung der SOEP-CoV-Studie 2020.

[CR39] Siewert J (2013). Herkunftsspezifische Unterschiede in der Kompetenzentwicklung: weil die Schule versagt? Untersuchungen zum Ferieneffekt in Deutschland.

[CR40] Tankard ME, Paluck EL (2016). Norm perception as a vehicle for social change. Social Issues and Policy Review.

[CR41] Walsh F (2016). Strenthening family resilience.

